# Antibacterial consumption in Romania in 2012: specific features and quality indicators for community usage

**DOI:** 10.1186/1471-2334-13-S1-O17

**Published:** 2013-12-16

**Authors:** Gabriel Adrian Popescu, Levente Mathyas, Corina Ciolan, Roxana Șerban, Adriana Pistol

**Affiliations:** 1 Carol Davila University of Medicine and Pharmacy, Bucharest, Romania; 2 IMS Health Romania, Bucharest, Romania; 3 National Institute of Public Health, Bucharest, Romania

## Background

The use of antimicrobials is the main cause for development of antimicrobial resistance. Many countries increasingly implement actions to control antimicrobial resistance through rational use. The information on national and local antimicrobial consumption can be an important source for planning rational use interventions and for monitoring progress towards a more prudent use of antibiotics. In Romania, the particularities of national antimicrobial consumption were never analyzed before 2012. We aimed to identify specific features of national antimicrobial consumption in Romania, and to calculate the quality indicators for community antibiotic usage.

## Methods

We performed an analysis of national antibiotics sales for 2012, provided by IMS Health Romania, a marketing research company using ATC classification; the consumption was measured in defined daily doses (DDD)/1000 inhabitants/day. Data were split into hospital sales and community sales. In order to evaluate the misuse of antibiotics, we calculated the values for the 12 European quality indicators of community usage of antibiotics as defined during the European Surveillance of Antimicrobial Consumption (ESAC) project.

## Results

Total consumption of antibacterials was 31.94 DDD/1000inh/d (third place in Europe in 2011). In community, the use of penicillins, cephalosporins, quinolones and sulfonamides exceeded the median for EU member states 1.78 to 2.46-fold; conversely, the use of tetracyclines, nitrofurantoin, fosfomycin was below the European level for these narrow spectrum antibiotics, 0.61-0.65-fold. The indicators place Romania in the latest positions of quality prescription in community (abuse and broader spectrum antibiotics used 8.14-fold more than narrow spectrum antibiotics). From community sales, 0.61 DDD/1000inh/d were parenteral antibiotics, more than one fourth of antibiotic consumption in hospitals, 2.18 DDD/1000inh/d. This level could place Romania in the fourth place among the 18 European states communicating hospital consumption for 2011; the most used antibiotics in hospital were penicillins, 0.86 DDD/1000inh/d, aminoglycosides, 0.44DDD/1000inh/d and cephalosporins, 0.40 DDD/1000inh/d.

## Conclusion

Our analysis indicates several issues: high level of consumption (abuse); low quality of antibiotic prescription with unnecessary consumption of broad spectrum antibiotics instead of narrow spectrum antibiotics (misuse); chaotic usage of antibiotics: parenteral treatments in community in the absence of outpatient parenteral antibiotic medical teams and oral therapy in unnecessary hospitalized patients. There is an urgent need to enhance the people education about risk of antibiotics, to enforce the legislation on antimicrobial sales, and to increase the quality of medical education for rational prescribing of antibiotics in the community and in hospitals (antimicrobial stewardship).

